# Unfolding New Roles for Guanine-Based Purines and Their Metabolizing Enzymes in Cancer and Aging Disorders

**DOI:** 10.3389/fphar.2021.653549

**Published:** 2021-04-16

**Authors:** P. Di Iorio, S. Beggiato, M. Ronci, C. B. Nedel, C. I. Tasca, M. Zuccarini

**Affiliations:** ^1^ Department of Medical, Oral and Biotechnological Sciences, University of Chieti-Pescara, Chieti, Italy; ^2^ Center for Advanced Studies and Technologies (CAST), University of Chieti-Pescara, Chieti, Italy; ^3^ Department of Pharmacy, University G. D'Annunzio Chieti, Chieti, Italy; ^4^ Laboratório de Biologia Celular de Gliomas, Programa de Pós-Graduação Em Biologia Celular e Do Desenvolvimento, Universidade Federal de Santa Catarina, Florianópolis, Brazil; ^5^ Laboratório de Neuroquímica-4, Programa de Pós-Graduação Em Bioquímica, Universidade Federal de Santa Catarina, Florianópolis, Brazil

**Keywords:** guanine-based purines, purine nucleoside phosphorylase, guanine deaminase, reactive oxygen species, cancer, aging disease

## Introduction

The ubiquitous purinergic system is composed by adenine- and guanine-based compounds, their converting enzymes (Yegutkin, 2014), and by the adenosine (P1) G protein-coupled receptors (GPCRs) and the nucleotide (P2) receptors, which are further classified into P2X_1-7_ ion channels and P2Y_1,2,4,6,11–14_ GPCRs (Burnstock, 2011). The activation of these receptors has been correlated to a number of patho-physiological conditions such as neurodegenerative diseases, cancer, ischemia and inflammation (Burnstock, 2018). Guanine-based purines (GBPs) are endogenous molecules comprising the nucleotides guanosine 5′-triphosphate (GTP), guanosine 5′-diphosphate (GDP) and guanosine 5′-monophosphate (GMP), the nucleoside guanosine (GUO) and the nucleobase guanine (GUA) (Schmidt et al., 2007).

GBPs inspired numerous studies in the late ‘90s, followed by a long period of sporadic works with a renewed research interest only in recent years. The reason why GBPs have been neglected likely relies on the lack of specific GBPs receptors able to confer them a real therapeutic potential.

GBPs share many structural and functional similarities with ABPs (Santos et al., 2006): they are released by many cell types, interconverted by soluble and membrane-bound ecto-enzymes and either taken up by selective nucleoside transporters or further metabolized up to the formation of uric acid (Zimmermann and Braun, 1996). Specifically, extracellular GUO is converted by purine nucleoside phosphorylase (PNP) to GUA that, in turn, is metabolized to xanthine (XAN) by guanine deaminase (GDA) (Yuan et al., 1999; Giuliani et al., 2016; Shek et al., 2019).

This brief work illustrates the most recent findings regarding GBPs and sheds light on the new therapeutic potential of Guanylates and their converting enzymes in cancer and age-related diseases.

## Roles of GBPs in the CNS

GBPs have been classically described as neuromodulators, playing neurotrophic and neuroprotective effects in the central nervous system (CNS) (Schmidt et al., 2007).

Indeed, there is a general consensus about GBPs behaving as a repair system upon brain injury in both *in vitro* and *in vivo* models (Lanznaster et al., 2016; Ribeiro et al., 2016). Accordingly, 1) higher extracellular levels of GBPs but not ABPs are detected in cultured astrocytes upon hypoxic or hypoglycaemic conditions (Ciccarelli et al., 1999) ii) GBPs, especially GUO, interferes with glutamatergic system by preventing glutamate excitotoxicity (Tasca et al., 2004; Lanznaster et al., 2017); iii) GBPs demonstrate anxiolytic, antidepressant and anticonvulsant effects (Tavares et al., 2008; Kovacs et al., 2015; Bettio et al., 2016; Frinchi et al., 2020); iv) GUO administration prevents NMDA-evoked neurotoxicity and apoptosis in hippocampal slices (Molz et al., 2008), inhibits the neurotoxin 6-hydroxydopamine (6-OHDA)-mediated apoptosis in a model of Parkinson’s disease (Giuliani et al., 2012b), induces neuroprotection in hippocampal slices subjected to oxygen/glucose deprivation (OGD) and ischemia (Ganzella et al., 2012; Dal-Cim et al., 2013); v) GUO stimulates neural stem cells and astrocyte proliferation (Ciccarelli et al., 2000; Su et al., 2013), as well as neurogenesis (Bau et al., 2005; Decker et al., 2007; Piermartiri et al., 2020); vi) GTP induces differentiation of C2C12 skeletal muscle cells and PC12 cells via Ca^2+^-activated K^+^ channel, upon phospholipase C (PLC)/inositol triphosphate (IP3)/diacylglycerol (DAG) activation (Gysbers and Rathbone, 1996; Guarnieri, Fanò et al., 2004; Pietrangelo, Fioretti et al., 2006; Mancinelli, Pietrangelo et al., 2012) vii) GUA improves learning and memory formation (Giuliani et al., 2012a; Zuccarini et al., 2018b).

The molecular mechanisms underlying GBPs-induced neuroprotection involve the activation of Phosphoinositide 3-kinase (PI3K)/Protein kinase B (PKB)/Glycogen Synthase Kinase3β (GSK3β), Protein kinase C (PKC), extracellular signal-regulated kinases (ERK) and Heme Oxygenase-1 (HO-1) signaling transduction pathways (Molz et al., 2011; Bellaver et al., 2015; Giuliani et al., 2015).

For an in-depth description of the pathophysiological roles of GBPs in the central nervous system we direct readers to these reviews (Di Liberto et al., 2016; Tasca et al., 2018; Mancinelli et al., 2020).

## GBPs in Aging Disorders

Reactive oxygen species (ROS) are involved in a wide number of age-related disorders in many organs and tissues. The end-products of GBPs metabolism, namely XAN and uric acid (UA), have been associated to ROS production and are, therefore, considered potential targets for anti-ageing strategies.

Thus, XAN-generating GDA has been evaluated in skin disorders such as Riehl’s melanosis (hyperpigmentary lesions of neck and face), psoriasis and, more in general, epidermal senescence (Kizaki et al., 1977). This enzyme is abundantly expressed in melasma, an hyperpigmentation caused by UV irradiation and inflammation (Noh et al., 2014). Upon chronic exposure to UVA or UVB radiations, GDA expressed in keratinocytes may trigger seborrheic keratosis by generating XAN, which is further metabolized to UA leading to the production of ROS and DNA damage (i.e., upregulation of *γ*-H2AX) (Cheong and Lee, 2020). ROS, in turn, can react with GUA and generate 8-oxo-7,8-dihydroguanine (8-oxoG) which is known to induce DNA damage and skin senescence (Valavanidis et al., 2009). Of note, GDA has also a direct role in skin lesions by interacting with several cytokines and growth factors, thus promoting melanogenesis (Jung et al., 2020).

Furthermore, in a murine model of lower urinary tract dysfunction (LUTD), 6 weeks-treatment with a PNP-inhibitor, 8-aminoguanine (8-AG), ameliorated LUTD symptoms (bladder structure and functions alterations and insensitivity) and reversed the age-associated up-regulation of several pro-apoptotic factors such as cleaved caspase-3, p16 and cleaved Poly (ADP-ribose) polymerase (PARP), a downstream effector of oxidative damage (Birder et al., 2020a). In addition, 8-AG decreased urinary levels of hypoxanthine but did not modify those of GUO. The protective effect of 8-AG in the urinary tract has been detected also in age-related urinary incontinence in female rats (Birder et al., 2020a). In this study, the PNP inhibitor reverted mitochondrial injury in urethra smooth and striated muscle and normalized oxidative and nitrosative markers.

## GBPs and Cancer

Over the last few years there has been a growing interest about the role of GBPs in cancer progression. As a matter of fact, GUA is not only a building block of DNA and RNA but also an extracellular signaling molecule involved in cell metabolism and proliferation.

DNA and RNA exhibit guanine (G)-rich sequences, namely GROs, able to self-assembly and form G-quadruplexes. G-quadruplex based aptamers showed therapeutic potential in several diseases such as HIV and cancer by targeting DNA promoter regions of oncogenes such as c-MYC, HIF-1α, VEGF (Collie and Parkinson, 2011). For example, the aptamer AS1411 was able to reduce tumor cell proliferation in human leukemic T cell lymphoblasts by targeting nucleolin, NF-kB and bcl-2 and is currently under phase II clinical trials for metastatic renal cell carcinoma (Bates et al., 1999; Soundararajan et al., 2008; Rosenberg et al., 2014). The cytotoxic activity of these nucleic acid drugs likely relied on the massive production of GBPs that would unbalance nucleotides/nucleosides ratio and subvert DNA repair mechanisms (Wang et al., 2019). Specifically, concerning the antiproliferative effect of guanine-based biomolecules, it has been demonstrated that in the leukemic T-cell lymphoblast the IC50 values were 14–18 μM (Zhang et al., 2015).

A recent study showed that the upregulation of inosinates and guanylates was associated with radiotherapy (RT)-resistance in glioblastoma multiforme (GBM) (Zhou et al., 2020). In this work, RT-sensitive cells (U118 MG, DBTRG-05MG, and GB-1) were exposed to nucleosides (cytidine, guanosine, uridine and thymidine at concentrations 80–240 μM) and showed a decreased RT ability to induce DNA double-stranded breaks (DSBs), thus promoting DNA repair and tumor cell survival. Interestingly, cell treatment with Mycophenolic acid (MPA) (10 μM), an inhibitor of inosine monophosphate dehydrogenase (IMPDH), an enzyme involved in *de novo* synthesis of guanine nucleotides, radiosensitized RT-resistant cell lines (U87 MG and A172). IMPDH inhibitors, responsible for increased IMP levels and reduced *de novo* synthesis of GTP and XMP have been developed as antiviral, antineoplastic (Cuny et al., 2017) and antimicrobial drugs (Shah and Kharkar, 2015). In line with these findings, Garozzo et al. previously reported that glioblastoma cell growth was inhibited by GUA, GUO and GMP with GI50 values of 44 ± 2.8, 137 ± 9.1 and 140 ± 10.2 µM, respectively (Garozzo et al., 2010).

In addition to the nucleobase GUA, a key role seems to be played by GUA-generating (PNP) and GUA-removing (GDA, Hypoxanthine Guanine Phosphoribosyltransferase-HGPRT) enzymes. PNP converts GUO into GUA and inosine (INO) into hypoxanthine (HYPO); GDA deaminates GUA into xanthine (XAN); HGPRT converts H YPO and GUA into IMP and GMP, respectively.

PNP inhibitors have been developed for the treatment of leukemia wherein they caused cell death via up-regulation of the apoptotic caspase-8, -9, and -3 and dGTP accumulation (Balakrishnan et al., 2006; Tong et al., 2009). PNP has also been employed in a gene-directed enzyme prodrug therapy (GDEPT), where the bacterial PNP metabolizes the substrate adenine analogue to the cytotoxic 2-Fluoroadenine (Balakrishnan et al., 2006; Afshar et al., 2009).

The chemotherapeutic effect of another purine nucleoside analogue, namely the deoxyguanosine analogue CNDAG, was reported in leukemias and linked to single- and double-strand breaks in DNA (Liu et al., 2020).

Furthermore, in leukemic cells lacking the expression of Sterile alpha motif and HD domain-containing protein 1 (SAMHD1), an enzyme degrading deoxyribonucleoside triphosphates (dNTPs), the administration of the PNP inhibitor, forodesine, caused cell apoptosis upon deoxyguanosine triphosphate (dGTP) overload (Kicska et al., 2001; Davenne and Rehwinkel, 2020). The antiproliferative activity of dGTP and deoxyguanosine (dGUO) was described in T- and B-lymphoid cells, although the molecular mechanism behind this effect remains as yet unclear (Chan, 1978).

In oncology, HGPRT plays a crucial role as it is considered a reporter gene able to detect somatic mutant cells and the related risk of cancer, therefore serving as cancer biomarker (Russo et al., 2004). Akin to PNP, HPRT has been used to activate the pro-drug deoxy-6-thioguanosine-5′-triphosphate which is responsible for cell apoptosis due to DNA mispairing (Yan et al., 2003). In a recent study, high levels of circulating uric acid in patients affected by gastric and pulmonary adenocarcinomas has been observed (Dumanskiy et al., 2020).

## Discussion

The lack of identified specific GBPs receptors able to provide a potential therapeutic target represents the main reason for the low interest in GBPs related research. Interestingly, a binding site for GUO has been already detected and it was recently reported that GUO would exert neuroprotection by interacting with A_1_R-A_2A_R heteromer (Traversa et al., 2002; Lanznaster et al., 2019). Moreover, several GTP binding sites were identified in excitable cells likely belonging to G_i/0_ protein-coupled receptor family and associated with [Ca^2+^]_i_ elevation (Pietrangelo et al., 2002).

The role of GBPs as neuromodulators is now well-documented (Tasca et al., 2018). GBPs and their converting enzymes have been studied in urinary dysfunctions and skin diseases (melasma, Riehl’s melanosis, seborrheic keratosis) where XAN- and UA-mediated ROS generation seems to promote DNA damage in age-related oxidative stress (Birder et al., 2020b). In the urinary tract, the accumulation of GUO and INO following PNP inhibition has a double protective role since it hampers the generation of urotoxic compounds and preserves the anti-inflammatory and protective nucleosides (Liu et al., 2009). As aging positively correlates with extracellular matrix (ECM) remodeling, the role of GDA, which is able to interact with ECM components (Zuccarini et al., 2018a), may be evaluated too.

A large body of evidence suggests a possible role of GBPs in cancer, with purine salvage pathway being the fuel of nucleotide pool maintenance and correct cell division. Several findings support the anti-proliferative effect of GUO, GUA and GMP in glioblastoma cells, prostate cancer cells, lung adenocarcinoma cells and myeloid leukemia cells (Garozzo et al., 2010; Zhang et al., 2015; Oliveira et al., 2017). The cytotoxic effect is due to their genotoxic activity that signals cell cycle arrest (Wang et al., 2019), although a recent study revealed that guanylates and inosinates would promote radio-therapy resistance and DNA breaks repair (Zhou et al., 2020).

Extracellular and intracellular GBPs amounts are related to the activity of GBPs converting enzymes, therefore their deficiency negatively correlates with GBPs effects. To reinforce this hypothesis, GDA gene knockout in dGUO insensitive HeLa cells induced cell response to the antiproliferative effect of dGUO. *Vice versa*, cell transfection with pCMV-Myc-GDA plasmid into the sensitive human embryonic kidney HEK293 cells prevented dGUO-mediated arrest at the S phase (Wang et al., 2019). More in general, the same authors suggested that in those cells were GDA expression was lower, GBPs antiproliferative effect resulted to be greater. A crucial role is played by PNP, HGPRT and IMPDH. These enzymes are part of *de novo* and salvage pathways and their modulation allow cells to meet metabolic needs and proliferate, as they ultimately lead to nucleotides synthesis (Tong et al., 2009).

The PNP inhibitor, forodesine, has shown therapeutic effects in the treatment of leukemias (Tong et al., 2009). It is worth to mention that most of these enzymes (i.e. GDA and IMPDH) exhibit a non-enzymatic activity; for example, SAMPHD1 and IMPDH can both interact with nucleic acids and are regulated by epigenetic mechanisms (Seamon et al., 2015). Importantly, the presence of GBPs metabolic enzymes both inside and outside the cell, makes it difficult to distinguish the origin of single nucleotides or nucleosides without inhibiting the respective enzymes.

In cancer therapy, GBPs demonstrated innovative therapeutic potential as they were used in gene-directed enzyme prodrug therapy (GDEPT) or in G-quadruplex based aptamers.

The greatest challenge about therapeutic applications of GBPs is represented by their ubiquitous expression and their involvement in pleiotropic circuits which may lead to unfavorable side effects in other organ/tissues. Therefore, it is extremely important to fine-tune purinergic signaling by controlling the expression/activity of these enzymes, with an eye on the complex network of simultaneously activated pathways.

**FIGURE 1:**
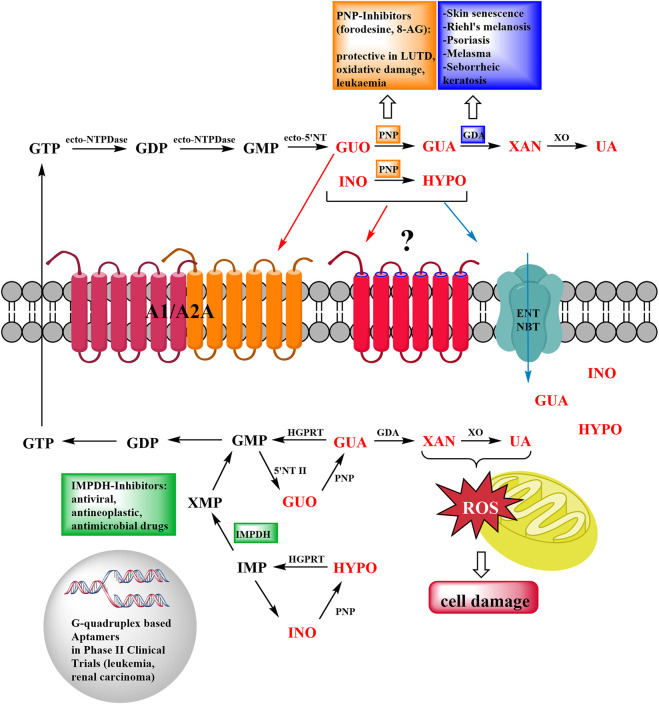
Schematic representation of the roles of Guanine-Based Purines and their metabolizing enzymes in cancer and aging disorders. At extracellular level, guanine-based nucleosides are metabolized up to the formation of uric acid (UA), they can also interact with adenosine (A1/A2A)/unknown metabotropic receptors, or enter the cell via specific equilibrative nucleoside transporters (ENT and NBT). At intracellular level, *de novo* and purine salvage pathways restore the purine nucleotide pool. Pharmacological manipulation of purine-converting enzymes demonstrated a therapeutic potential in LUTD, oxidative damage and leukemia (PNP), skin disorders (GDA), viral and microbial infections (IMPDH). Ecto-NTPDase: Ecto-nucleoside Triphosphate diphosphohydrolase; PNP: Purine Nucleoside phosphorylase; GDA: Guanine deaminase; XO: Xantine oxydase; HGPRT: Hypoxanthine-guanine phosphoribosyltransferase; 5′-NT II: Cytosolic 5′-nucleotidase II; IMPDH: Inosine Monophosphate dehydrogenase.

Taken together, these recent findings unravel the high translational potential of GBPs not only in neuromodulation but also in age-related diseases and cancer (Figure 1), where an unbalance in nucleotides/nucleosides/nucleobase ratio become crucially important as it directs cells toward senescence/apoptotic processes or uncontrolled cell proliferation.
